# Emergency response strategy for containing COVID-19 within a psychiatric specialty hospital in the epicenter of the COVID-19 epidemic in China

**DOI:** 10.1038/s41398-020-00959-3

**Published:** 2020-08-04

**Authors:** Jun Ma, Hao Zhong, Min Jiang, Kuan Zeng, Baoliang Zhong, Lianzhong Liu, Xuebing Liu

**Affiliations:** Affiliated Wuhan Mental Health Center, Tongji Medical College of Huazhong University of Science & Technology, Wuhan, China

**Keywords:** Psychiatric disorders, Scientific community

## Abstract

Coronavirus disease 2019 (COVID-19) has been recognized as a global pandemic, and psychiatric institutions located in the epicenter of the epidemic in China are facing severe challenges in fighting the epidemic. This article presents the accumulated experience of the authors during the process of combating COVID-19 in a psychiatric hospital. The aim of this article is to provide a reference for psychiatric specialty hospitals and institutions that treat large populations of chronically ill patients in other parts of the world.

## Introduction

Coronavirus disease 2019 (COVID-19) is an acute respiratory infectious disease caused by severe acute respiratory syndrome coronavirus 2 (SARS-CoV-2)^1^. The disease was first reported in Wuhan City, Hubei Province, in December 2019^2^. The rapid spread of COVID-19 and its serious consequences pose severe challenges to public health in China and other countries worldwide^3,4^. Similar to other medical institutions, psychiatric specialty hospitals located at the epicenter of the COVID-19 pandemic are also poorly prepared^5^, and patients and medical staff in mental health institutions have been infected in some areas^6,7^. Because SARS-CoV-2 is highly infectious, and the general population is susceptible, it is difficult to effectively prevent the spread of COVID-19 based on the current instruments, medical staff, and management modes of mental health institutions. Therefore, mental health institutions in areas severely affected by the COVID-19 epidemic face serious challenges^8^.

The authors’ work unit is the largest Grade A psychiatric specialty hospital in China’s COVID-19 epidemic-affected area. On January 30, 2020, the institution closed and isolated the floor of the clinical department where an outbreak occurred. On February 13, the Wuhan epidemic prevention and control command instructed that an isolation ward should be established to treat patients with mental illnesses with confirmed or suspected of COVID-19 infection. In accordance with the principles and requirements of the supervising department of “receiving all patients” and having “sufficient sickbeds for potential patients,” within 7 days, a branch of our hospital, which can accommodate 500 isolated patients, was transformed into an isolation ward. The authors were among the frontline staff and were engaged in the treatment of psychiatric patients infected with COVID-19. This paper shares the experiences of managing COVID-19 and preventing cross-infection at a psychiatric specialty hospital. We hope to help psychiatric hospitals and other chronic disease institutions in other parts of the world that also face COVID-19 to reduce and control infection.

## Response plan for COVID-19 in mental health institutions

### Ward reconstruction

#### Structural reconstruction of wards

Hospitalization wards for patients with severe mental illness are generally closed or semiclosed. This arrangement cannot prevent the spread of COVID-19, and it is difficult to effectively isolate and treat infected or suspected patients. Therefore, ward reconstruction is an essential step.

The existing ward layout can be retrofitted based on the current ward layout of infectious disease hospitals. Reconstructed wards have a structure consisting of “three areas and two access points.” The “three areas” include a clean area, a semicontaminated area, and a contaminated area, and the “two access points” include a contaminated access for medical waste and the transfer of infected patients and a clean access, which allows access for medical personnel and daily clinical work. Under the premise that no specific therapeutic drugs or vaccines exist for COVID-19, isolation is still the most effective means of containing COVID-19 in institutions.

#### Reallocation of ward functions

Before the COVID-19 outbreak, psychiatric wards were generally designed according to the characteristics of conventional psychiatric services, comprising a dementia ward, a depression ward, and a substance dependence ward, for example. During the outbreak, all impairment-specific settings were replanned to allow for the most effective prevention and control of COVID-19, and the functions of the psychiatric wards were reallocated according to the following plan^9^: (1) a ward for confirmed COVID-19 patients: this ward is used for the treatment of COVID-19 patients, and the patients are classified and managed according to disease severity; secondary protections should be implemented. (2) A ward for suspected COVID-19 patients: this ward is used for patients with negative nucleic acid test results but with clinical and imaging manifestations similar to those of COVID-19 patients; secondary protections should be implemented. (3) An isolation observation ward: this ward is used for the temporary isolation and observation of patients who are newly admitted to the hospital and have no symptoms of pulmonary infection; secondary protections should be implemented. During this isolation period, patients are screened, confirmed patients are transferred to the ward for confirmed COVID-19 patients, suspected patients are transferred to the ward for suspected COVID-19 patients, and clean (uninfected) patients are treated in this ward. (4) A rehabilitation ward: this ward is used for the medical isolation and observation of patients who have been cured or meet the discharge criteria during the rehabilitation period; primary protection should be implemented. (5) A general ward: this ward is used for the centralized and closed management of patients who have not been affected by COVID-19; primary protection should be implemented. The admission and treatment of new patients is not allowed, and medical staff should be managed in an “inpatient” manner, with regular shift changes and restrictions of movement to prevent cross-infection.

### Optimization of resource allocation

Due to the lack of awareness of virus transmission routes, in the early stage of a COVID-19 outbreak, the following issues will inevitably occur in institutions: (1) serious staff shortages due to the infection and isolation of medical staff; and (2) shortages of protective equipment. If these problems are not addressed in a timely manner, they can lead to continued aggravation of a COVID-19 outbreak at a hospital.

It is urgent to utilize all available resources for self-help according to the following recommendations: (1) shortages of first-line medical staff can be reduced by reorganizing wards, sending non-frontline health workers to work in the ward, and recruiting new medical staff. (2) If nosocomial infection occurs in the early stage of the outbreak, the hospital should be temporarily closed and stop admitting new patients. After the nosocomial infection is under control, diagnosis and treatment procedures should be reorganized in accordance with the requirements for preventing and treating infectious diseases. (3) Facing the difficulties of a shortage of protective equipment hospitals, it is necessary to actively establish a wide range of social connections, accept social donations, strengthen communication and coordination with the government, and request material assistance.

### Ensure good liaison consultation, admission, and referral of patients

**Fig. 1:**
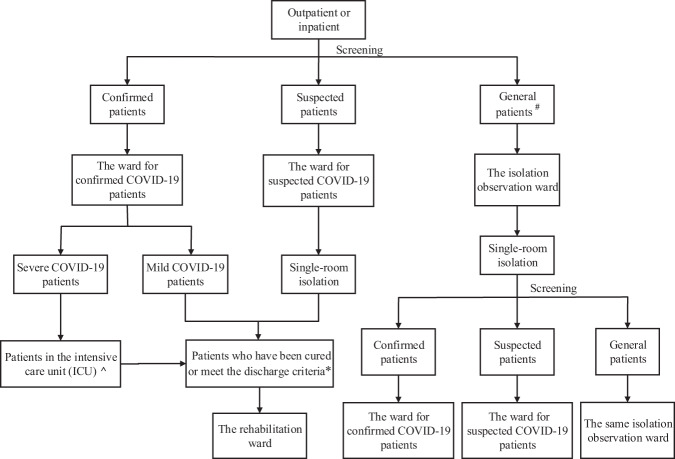
Patient admission and referral process. A hash (#) represents general patients; patients without symptoms and signs of infection, including those with asymptomatic infections, close contact with COVID-19 patients, and new admissions. An up arrowhead (^) represents Wuhan Jinyintan Hospital, which was the designated hospital for local severe COVID-19 cases. An asterisk (*) represents the criteria for cured and discharged patients are defined in the *Diagnosis and Treatment of COVID-19* (7th trial edition)^10^.

Liaison consultation, admission, and referral of patients according to their infection conditions are important factors in determining the success or failure of the fight against COVID-19. Hospital management must establish a team of experts to assess COVID-19 patients according to the guidelines for the diagnosis and treatment of COVID-19 in China^10^ and assign patients to inpatient wards according to their conditions (Fig. 1). Regarding the expert team, in addition to professional psychiatric medical staff, infectious disease experts are also responsible for guiding the transformation of wards and the control of in-hospital transmission of the epidemic. Respiratory disease experts are responsible for assessing the severity of lung infections, providing in-depth guidance on the treatment of infections, and ultimately deciding whether a patient needs to be referred for treatment.

### Prevention and effective control of nosocomial infections

The importance of controlling and preventing nosocomial infections cannot be overemphasized in the fight against COVID-19. In psychiatric specialty institutions, psychiatric staff have insufficient experience in the prevention and control of nosocomial infections. The following efforts must be made under the guidance of an infectious disease physician:

1. Effectively redesign the overall layout of the hospital for the prevention and control of COVID-19: the entire hospital, including the outpatient and inpatient wards, must have clean and contaminated accesses to prevent cross-infection.
2. Provide emergency training of staff for the prevention and control of nosocomial infections: this training includes performing hand hygiene, wearing and removing protective clothing, division of functional areas, protection levels and protective measures in different areas, disinfection of different environments, daily life of staff, methods to transfer patients with different diseases, methods to manage patients in different wards, and methods to dispose of medical waste.
3. Provide training in the prevention and control of nosocomial infections to workers in the ward, including doctors, nurses, and cleaning staff, to ensure that each staff member can perform standardized operations.

## Effectiveness of the prevention and control of the COVID-19 outbreak

In mid-January 2020, during the early stage of the outbreak, COVID-19 infections of patients and medical workers occurred in some inpatient wards in our hospital, and the situation was serious. Our hospital immediately stopped admitting and treating new patients and began reconstructing the wards, changing the functions of the wards, standardizing the work process, and training the medical staff to increase their awareness of nosocomial infections. After implementing these measures, the sources of infection were effectively controlled, transmission routes were blocked, cross-infection was successfully prevented, and the effectiveness of the prevention and control of the COVID-19 outbreak was satisfactory. In late February, the COVID-19 outbreak in the hospital was quickly controlled, and inpatient services for patients with mental illnesses resumed at the hospital. On April 22, all patients in our hospital were negative according to SARS-CoV-2 nucleic acid detection, and the outbreak was considered to be “cleared”; on the 26th of that month, infected patients in Wuhan City, Hubei Province, were “cleared,” and the supervising authority announced the removal of the isolation ward (an isolation ward with a capacity of 120 patients remains to accommodate homeless patients during their convalescence period as well as sporadic infections that may occur). In early May, the outpatient department of the hospital reopened, and patients were admitted to the inpatient ward as before. The hospital gradually returned to its prior mode from before the COVID-19 pandemic.

## Lessons learned

1. Early detection, diagnosis, isolation, and treatment should be implemented to slow or block the spread of COVID-19.
2. If an outbreak of nosocomial infection occurs, the transformation from general wards to infectious wards should be the primary task. The inpatient ward should be closed and redesigned as soon as possible, including changing its function and the standardized workflow.
3. During the early stage of a COVID-19 outbreak, hospital workers and administrators should increase their vigilance and actively seek help from the government and society to ensure an adequate supply of manpower, material resources, financial resources, and protective equipment in preparation to manage the COVID-19 pandemic.
4. In the face of an infectious disease that spreads quickly, medical isolation is an important measure to control the spread of the epidemic in hospitals. Patient transfers should be reduced as much as possible, including confirmed patients, suspected patients, and even clean patients.
5. When centralized management is carried out for confirmed patients, suspected patients, and clean patients, they should be transferred individually, and the transfer channels should be strictly differentiated. After transfer, the channels should be effectively disinfected in a timely manner.
6. Control of nosocomial infection is essential for the success of COVID-19 prevention. Successful efforts can effectively block the spread of COVID-19 in hospitals. Preventive efforts must be performed as early and as quickly as possible. In the future, prevention work should be a routine part of the daily schedule of the hospital rather than a last-minute effort.
